# Three-Dimensional Reduced Graphene Oxide/Poly(3,4-Ethylenedioxythiophene) Composite Open Network Architectures for Microsupercapacitors

**DOI:** 10.1186/s11671-019-3098-4

**Published:** 2019-08-06

**Authors:** Xiling Mao, Xin He, Jianhua Xu, Wenyao Yang, Hao Liu, Yajie Yang, Yujiu Zhou

**Affiliations:** 1 State Key Laboratory of Electronic Thin Films and Integrated Devices, University of Electronic Science and Technology of China (UESTC), Chengdu, 610054 People’s Republic of China; 2 School of Electrical and Electronic Engineering, Engineering Research Center of Electronic Information Technology and Application, Chongqing University of Arts and Sciences, Chongqing, 402160 People’s Republic of China

**Keywords:** Vapor phase polymerization, Microsupercapacitors, Energy storage, Array devices

## Abstract

The three-dimensional (3D) porous nanostructures have shown attractive promise for flexible microsupercapacitors due to their merits of more exposed electrochemical active sites, higher ion diffusion coefficient, and lower charge-transfer resistance. Herein, a highly opened 3D network of reduced graphene oxide/poly(3,4-ethylenedioxythiophene) (rGO/PEDOT) was constructed through the laser-assisted treatment and in situ vapor phase polymerization methods, which can be employed with gel electrolyte to prepare flexible microsupercapacitors, without conductive additives, polymer binder, separator, or complex processing. These porous open network structures endow the obtained microsupercapacitors with a maximum specific capacitance (35.12 F cm^−3^ at 80 mA cm^−3^), the corresponding energy density up to 4.876 mWh cm^−3^, remarkable cycling stability (with only about 9.8% loss after 4000 cycles), and excellent coulombic efficiency, which are comparable with most previous reported rGO-based microsupercapacitors. Additionally, the microsupercapacitors connected in series/parallel have been conveniently fabricated, followed by being integrated with solar cells as efficient energy harvesting and storage systems. Moreover, the working voltage or energy density of microsupercapacitors array can be easily tailored according to the practical requirements and this work provides a promising approach to prepare high-performance flexible micro-energy device applied in the wearable electronics accordingly.

## Introduction

These dramatically pervasive smart microelectronic devices such as wireless sensor networks for on-line monitoring, biomedical implants for human health care, and real-time tracking chips, have led to the growing demands of lightweight, flexible, low cost, and highly efficient micro-scale energy storage devices [1–3]. Currently, commercially available thin-film and 3D micro-batteries, as the major micro-power sources, often suffer from poor rate performance, abrupt failure, and safety concerns. In comparison, interdigital microsupercapacitors (MSCs) are the dominant candidates in self-powered microelectronic devices because of their competitive power density, excellent safety, and superior rate capability, as well as long operational lifespan [4–6]. As one of the common configurations, the two-dimensional (2D) interdigital MSCs are widely employed due to their greatly reduced thickness and relatively high-power supply compared with a commercial supercapacitor. In general, the 2D interdigital MSCs need thicker microelectrodes to meet energy demand in a given footprint, whereas the thick microelectrodes may offer poor accessibility to electrolyte, insufficient charge transport, and increasing electron/ion diffusion distances, resulting in degrading the capacity and rate performance [1]. Thus, it is still challenging to increase their energy/power densities without compromising other electrochemical characteristics simultaneously in a limited footprint area.

Notably, the 3D opened network architecture has attracted great attention, owing to the merits of higher specific surface area, rapid ion transport, and buffer volume change during GCD cycle tests [7]. Until now, majority of approaches have been applied to synthesize 3D opened network microelectrodes including colloidal template [8, 9], hard template [10, 11], hydrothermal method [7, 12], and deposition on 3D substrates [4, 13, 14]. However, these conventional fabrication techniques often need the toxic agents, harsh synthetic conditions, or complex preparation technique, resulting in difficulties to obtain the cost-effective, large-scale, and environmentally friendly devices for commercial application. To overcome these obstacles, tremendous efforts have been devoted to exploring new strategies to manufacture 3D opened network MSCs efficiently. Impressively, the readily scalable and low-cost laser-assisted treatment [15–17], which can design the treating circuit at precise locations by software control to form the desired patterns without additional external wire, has attracted widespread attention to fabricate in-plane opened network MSCs. Additionally, the vapor phase polymerization (VPP) method involves the polymerization of precursor in the vapor phase onto the surface of oxidant [18], and it is readily tailored to prepare any desired patterns on varied substrates conveniently. More importantly, the VPP method is obvious superiority compared with chemical vapor deposition (CVD) [19], electrochemical deposition [20, 21], and in situ chemical polymerization [22], because it can get rid of the constraints of specialized vacuum equipment, electrolytic deposition device, or solvent processing.

As the key component for in-plane interdigital MSCs, microelectrode materials with the high surface areas, good hydrophilicity, and excellent ion intercalation behavior should be explored to improve their energy storage performance. Especially, rGO has aroused widespread attention due to its low-cost and abundant raw material (graphite), high electrical conductivity, and high surface area (2630 m^2^ g^−1^) [1]. However, the rGO-based MSCs generally release a relatively low specific capacitance, and the charges only accumulate at the interface between the electrode and electrolyte, resulting from the electrochemical double-layer capacitance energy storage mechanism [23]. In addition, conducting polymers such as PEDOT and their derivatives, which rely on fast and reversible faradaic redox reactions at the surface and/or in the bulk [24], have been intensively investigated as pseudo-capacitor electrodes because of their low toxicity, high conductivity, stable doped form, and low cost. Consequently, the rGO made by laser-assisted treatment and PEDOT via readily scalable VPP method are the optimal combination to fabricate opened network rGO/PEDOT microelectrodes.

Herein, we constitute the high-performance all-solid-state flexible microsupercapacitors based on interdigital rGO/PEDOT composite. Notably, the interconnected network rGO derived from graphene oxide (GO) by laser-assisted treatment is adopted as a conductive framework, ascribing to its merits of the tuning the surface morphology, controlling the desired pattern at precise locations, enhancing electrolyte wetting or diffusion kinetics. Then, the 3D open porous PEDOT prepared by VPP method can provide the accessibility to the electrolyte ions, shorter planar ionic diffusion path, and more electrochemical active sites. The in-plane interdigital MSCs employed these obtained rGO/PEDOT microelectrodes with PVA/H_3_PO_4_ gel electrolyte showed a maximum specific capacitance of 35.12 F cm^−3^, the energy density of 4.876 mWh cm^−3^ at 40 mW cm^−3^ under the current density of 80 mA cm^−3^, and outstanding cycling stability after 4000 cycles. Additionally, the MSCs connected in series/parallel had been constructed to power the red light-emitting diode (LED) light about 100 s when fully charged. Therefore, this work provides a facile way to prepare coplanar interdigital MSCs as micro-storage sources for next-generation highly integrated portable microelectronic devices where high capacity per limited footprint is critical.

## Experimental Methods

### Materials

The 3,4-ethylenedioxythiophene (EDOT) monomers were provided by Bayer AG. Iron (III) p-toluenesulfonate (Fe(PTS)_3_) and polyvinyl alcohol (PVA) powders were bought from Sigma-Aldrich. The GO nanosheets were purchased from Pioneer Nanomaterials Technology. Polyethylene terephthalate (PET) substrate, sodium dodecyl benzenesulfonate (NaDBS), phosphoric acid (H_3_PO_4_), acetone, ethanol, and other reagents were provided by Kelon Chemical Industry Co., Ltd. All the chemical reagents were used without further treatment. The program controlled the 788 nm infrared laser (maximum power output = 5 mW) inside a consumer-grade LightScribe optical drive unit by periodically pulsing an objective lens assembly, and the desired pattern can be prepared rapidly at precise locations. All the experiments were performed under ambient conditions.

### Synthesis of 3D Opened Network rGO/PEDOT Interdigital Electrodes

**Fig. 1:**
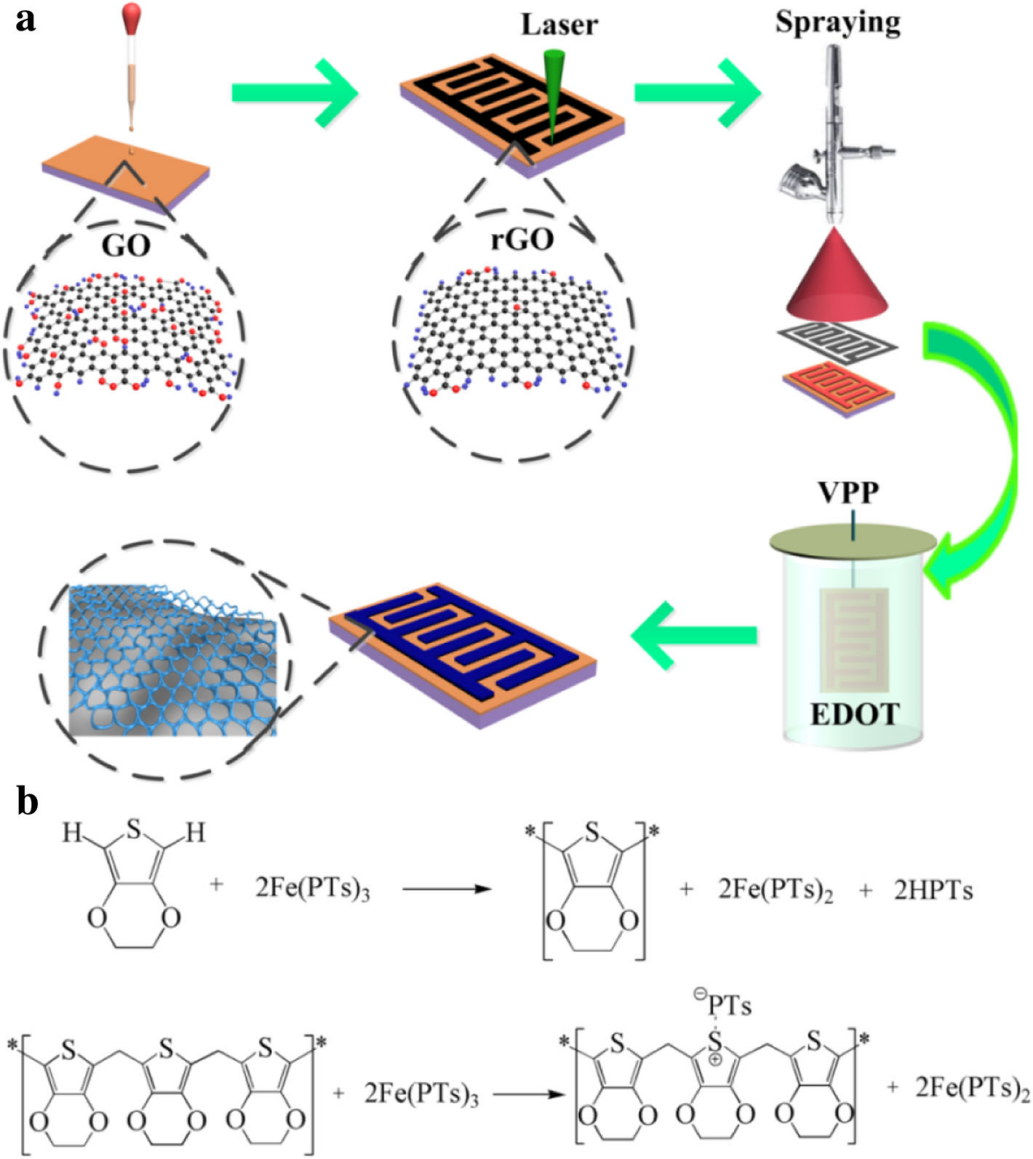
**a** Schematic illustration of the fabrication of rGO/PEDOT interdigitated electrodes. **b** The polymerization mechanism of PEDOT

Figure 1a shows a schematic illustration of the fabrication of rGO/PEDOT interdigital electrodes. In a typical procedure, a flexible polyethylene terephthalate (PET) substrate was cut into a square piece (2 cm × 2 cm) and was washed with ethanol, acetone, and deionized water several times, respectively. The GO was synthesized using a modified Hummer’s method [25], and the homogeneous 2% GO dispersion in deionized water was prepared by ultrasonic dispersion [26]. Then, the GO film was deposited on the PET substrate and allowed to dry about 24 h under ambient conditions. Subsequently, the GO-coated PET was put into the consumer-grade LightScribe optical drive unit for laser patterning, and 500 μs exposure duration of each voxel was adopted using the 788 nm infrared laser (power output about 100 mW). After setting the desired patterns into a computerized commercial drive, the conductive rGO interdigital electrodes were prepared rapidly at precise locations by periodically pulsing on the insulating GO film about 30 min, as our previously reported [21, 27].

Prior to fabricating the porous PEDOT by VPP, the as-prepared rGO sample was treated by 0.5 mg mL^−1^ NaDBS aqueous solution as the surfactant for 20 min and then baked at 80 °C about 5 min. The molar ratio 1:1 of Fe(PTS)_3_ to isopropanol was prepared as oxidant solution by magnetic stirring, which was then selectively deposited on the as-treated rGO interdigital electrodes with a mask by the spraying method. Subsequently, the obtained sample was positioned at the center of a small chamber containing 100 μL EDOT monomers, and the whole device was heated in the vacuum desiccator. The polymerization mechanism of PEDOT by VPP was shown in Fig. 1b. After applying the above samples exposed in the EDOT vapor at 30 °C, 50 °C, 80 °C, and 100 °C for 30 min, these highly 3D opened network rGO/PEDOT microelectrodes were fabricated, noting as rGO/PEDOT-30, rGO/PEDOT-50, rGO/PEDOT-80, and rGO/PEDOT-100, respectively. Additionally, the pristine rGO interdigital electrodes were also prepared as a comparison.

### Assembly of Highly Opened Network rGO/PEDOT-Based Flexible Interdigital MSCs

Typically, the PVA powder (1 g) was dissolved into deionized water (10 mL) at 90 °C for 2 h under vigorous stirring, then the H_3_PO_4_ (2 mL) was gradually added under slowly stirring at ambient temperature until forming a transparently jelly-like solution, and the PVA/H_3_PO_4_ gel electrolyte was successfully prepared. In addition, a metal coat was firstly covered on the surface of electrodes by sputtering as current collector, and the PVA/H_3_PO_4_ gel electrolyte was drop by drop covered onto the rGO/PEDOT interdigital electrodes. Subsequently, the device was soaked at room temperature for 10 h to ensure completely wet and evaporation of any excess water. Finally, the all-solid-state MSCs were successfully assembled.

### Characterization and Measurement

The morphologies, microstructural, and component characterizations were performed by scanning electron microscope (SEM), Fourier transform infrared spectroscopy (FTIR), and X-ray photoelectron spectroscopy (XPS). Additionally, the electrochemical properties (cyclic voltammetry (CV), galvanostatic charge/discharge (GCD), and electrochemical impedance spectroscopy (EIS) of flexible planar MSCs were examined by two-electrode cell on a CHI660D electrochemical workstation (Chen Hua, Shanghai) under ambient temperature.

The volumetric specific capacitances *C*_v_ (F cm^−3^), energy densities *W* (Wh cm^−3^), and power densities *P* (W cm^−3^) were calculated from the GCD curves at different current densities using the following Eqs. (1)–(3): [13, 17, 24].

1 $$ {C}_{\mathrm{v}}=\frac{I\times \Delta  t}{V\times \Delta  E} $$

2 $$ W=\frac{C_{\mathrm{v}}\times {\Delta  E}^2}{2\times 3600} $$

3 $$ P=\frac{W}{\Delta  t} $$

where *I* is the discharge current (A); *Δt* is the discharge time (s); *V* is the stack volume (cm^3^) which includes the combined volume of the active material, current collector, and the gap between the electrodes; and *ΔE* is the potential window (V).

## Results and Discussion

### Morphology and Structure of the GO, rGO, and rGO/PEDOT Electrode Materials

**Fig. 2:**
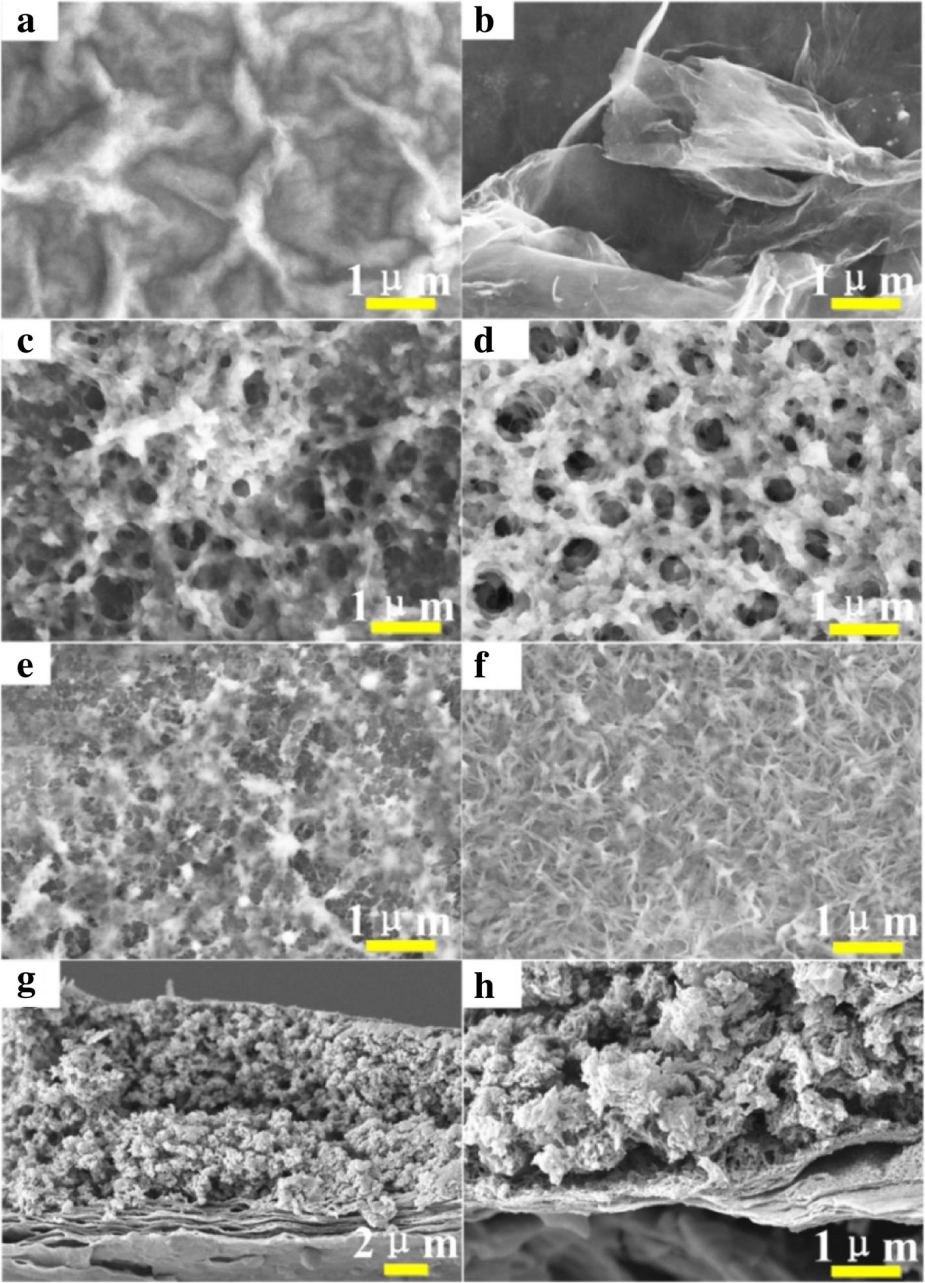
The typical SEM images of top view for **a** GO, **b** rGO, **c** rGO/PEDOT-30, **d** rGO/PEDOT-50, **e** rGO/PEDOT-80, and **f** rGO/PEDOT-100. The cross-section images **g** and **h** of rGO/PEDOT-50

The morphologies of the GO, rGO, and rGO/PEDOT were investigated by SEM shown in Fig. 2. Firstly, the richly 3D wrinkle-like rGO (Fig. 2b) derived from GO sheets (Fig. 2a) by the laser treating process can provide abundant charge carrier sites and allow ions to readily access or penetrate into their internal surfaces between the electrode and electrolyte. Importantly, these synergistic interactions of rGO and PEDOT networks are beneficial for shortening the diffusion distance and facilitating ion transport to achieve excellent energy storage properties [28]. Furthermore, the top view and the cross-section images of the four rGO/PEDOT samples polymerized at 30 °C, 50 °C, 80 °C, or 100 °C by VPP reveal various porous configurations (Fig. 2c–h). Additionally, compared with other three rGO/PEDOT samples, the rGO/PEDOT-50 (Fig. 2d) shows the homogeneous porous network structure, which is beneficial to improve the specific surface area and abundant conductive path. This may be owing to the appropriately slow evaporation of the side product acid and low-film growth rate at 50 °C, which are beneficial to endow homogeneous porous network during the polymerization process. Moreover, the higher polymerization temperatures (such as 80 °C, 100 °C) may tend to greater heterogeneous nucleation to form the dense flat morphology because of the higher EDOT vapor concentration and faster reaction rates, while the polymerization temperatures at 30 °C is too low to inadequate polyreaction [29, 30].

**Fig. 3:**
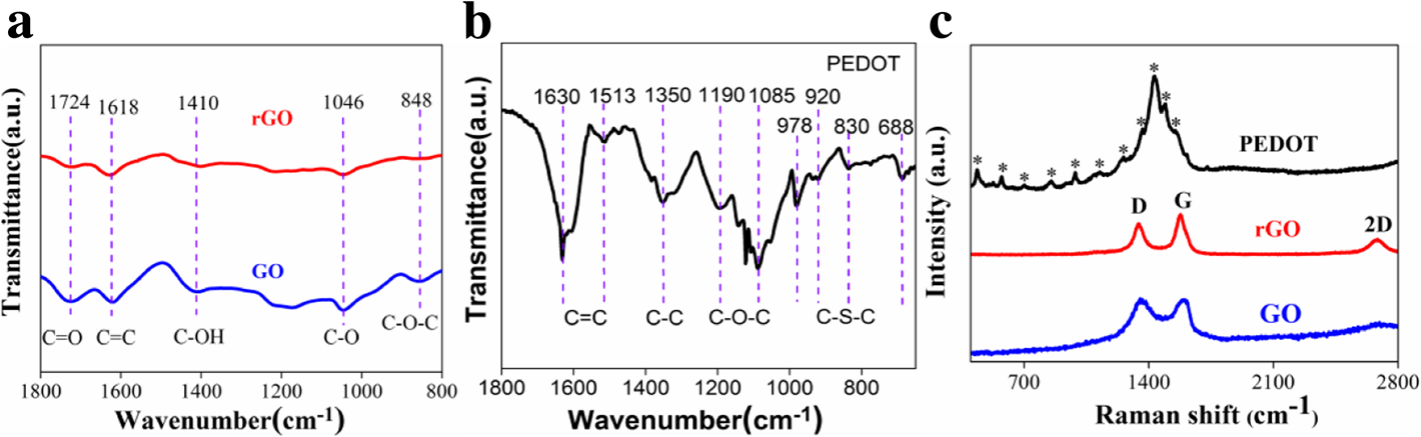
FTIR spectra of **a** GO, rGO, and **b** PEDOT. **c** Raman spectra of GO, rGO, and PEDOT

The FTIR and Raman measurements of GO, rGO, and PEDOT were also performed to characterize the changes of chemical compositions in Fig. 3. The typical characteristic peaks of GO (Fig. 3a) exhibit the C=O (1724 cm^−1^), C=C (1618 cm^−1^), C–OH (1410 cm^−1^), C–O (1046 cm^−1^), and C–O–C (848 cm^−1^). After laser-assisted treatment, all the absorption peaks of oxygen-containing functional groups were nearly removed, indicating the successful preparation of rGO (Fig. 3a). Additionally, these characteristic peaks of PEDOT, such as the asymmetric C=C stretching peak (1630, 1513 cm^−1^) [31], the C–C stretching mode (1350 cm^−1^), C–O–C deformation peak (1190, 1085 cm^−1^), symmetric C–S–C deformation peak (978, 920, 830, and 688 cm^−1^) [32] could be observed in Fig. 3b, further confirming the existence of PEDOT. Therefore, these FTIR spectra confirm the successful preparation of rGO/PEDOT composite through the laser reduction and VPP methods.

Figure 3c shows the Raman spectra of GO, rGO, and PEDOT. The *D* band is triggered by defects in the hexagonal carton materials, and the *G* band arose from the graphitic carbon (E2g mode). Furthermore, the intensity ratio of the *D* and *G* bands (*I*_*D*_/*I*_*G*_) is widely used to evaluate the disordered and ordered domains of graphene [27]. Obviously, the *D* (1359 cm^−1^) and *G* (1595 cm^−1^) bands of GO and rGO are both present in Fig. 3c, and the *I*_*D*_/*I*_*G*_ of GO and rGO are 1.02 and 0.92, respectively. The *I*_*D*_/*I*_*G*_ of rGO is lower comparing with GO, signifying the less defects of rGO after laser-induced treatment. More importantly, the prominent 2D peak (2687 cm^−1^) appears in the Raman spectra of rGO, further verifying the presence of few layer graphene [33]. Additionally, the 1548 and 1487 cm^−1^ peaks (*C*_*α*_ = *C*_*β*_), 1433 cm^−1^ peak (*C*_*α*_ = *C*_*β*_ (−O)), 1365 cm^−1^ peak (*C*_*α*_–*C*_*β*_), 1258 cm^−1^ peak (*C*_*α*_–*C*_*α*_), 1130 cm^−1^ peak (C–O–C), 988 cm^−1^ and 854 cm^−1^ peaks (C–S–C), and 442 cm^−1^ peak (S–O) are clearly observed in Raman spectra of PEDOT, which are in good accordance with the reported literature [34]. The above analyses evidently demonstrate the successful preparation of rGO and PEDOT.

**Fig. 4:**
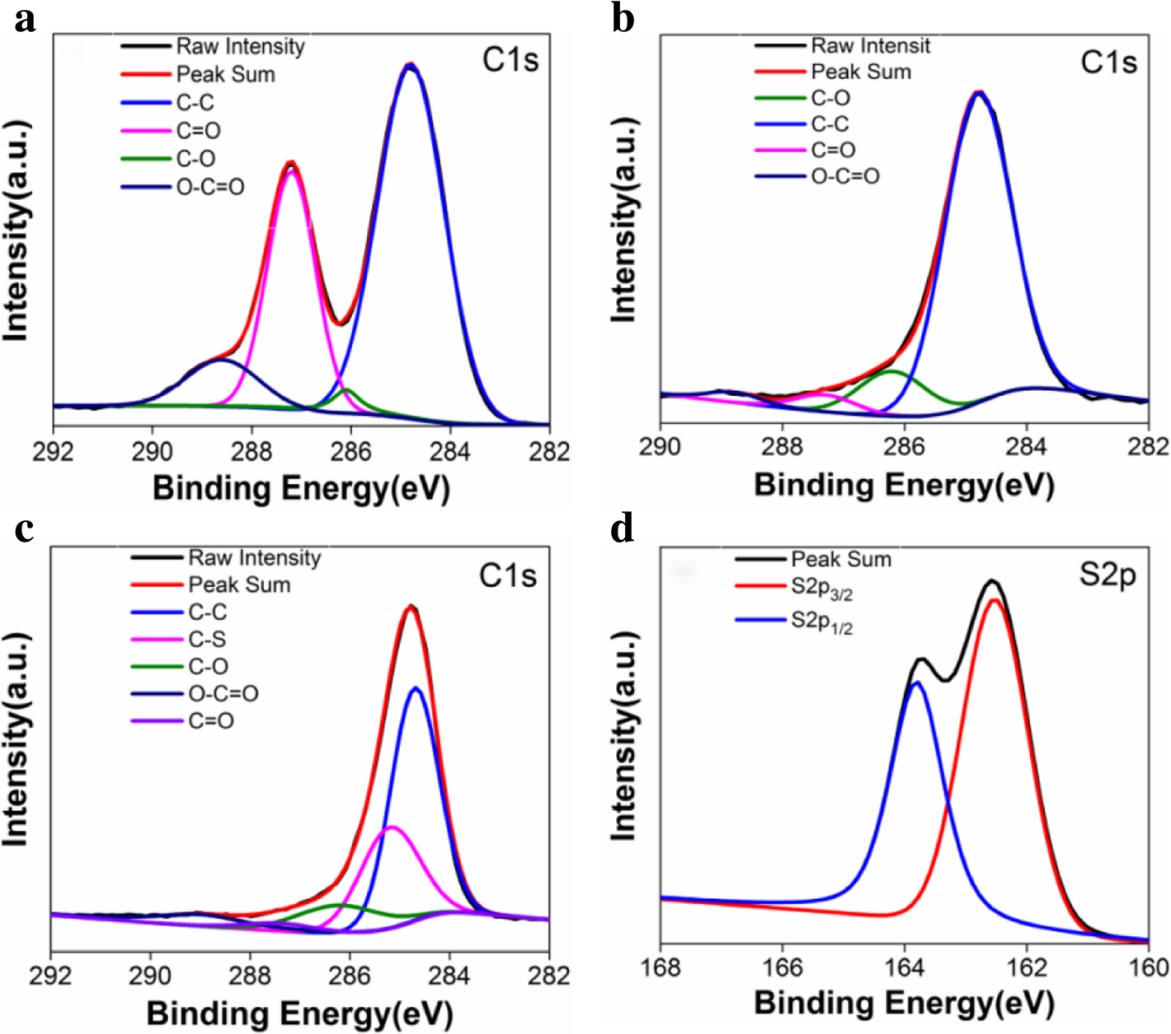
The survey XPS spectrum for C1*s* peaks of **a** GO, **b** rGO, and **c** rGO/PEDOT and **d** S2*p* peak of rGO/PEDOT

The XPS spectrum analysis of rGO/PEDOT, GO, and rGO were performed to monitor the oxygen functionalities (Fig. 4). The C1s spectrum of GO (Fig. 4a) and rGO (Fig. 4b) are resolved into multiple peaks of C–C (284.8 eV), C=O (287.3 eV), C–O (286.2 eV), and O–C=O (288.5 eV). In contrast to GO, the significant removal of oxygen-containing functional groups (C=O and O–C=O) and an overall increase in the C–C sp^2^ carbon peak of rGO point to an efficient deoxygenation process as well as restoration *π*-conjugated structure, resulting in a higher electrical conductivity after laser treating, these results also agree with the previous reports [35, 36]. The presence of C–S bonds (285.3 eV) in Fig. 4c further confirms the successful synthesis of PEDOT on the rGO. Moreover, Fig. 4d shows the S2*p* peak of rGO/PEDOT cracked into S2*p*3/2 (162.6 eV) and S2*p*1/2 (163.8 eV) doublets with a corresponding 1.2 eV separation, originating from the S atom bonded onto the thiophene ring structure in the chains of PEDOT [19, 32, 37].

### Electrochemical Behavior of the Flexible MSCs with Opened Network rGO/PEDOT

**Fig. 5:**
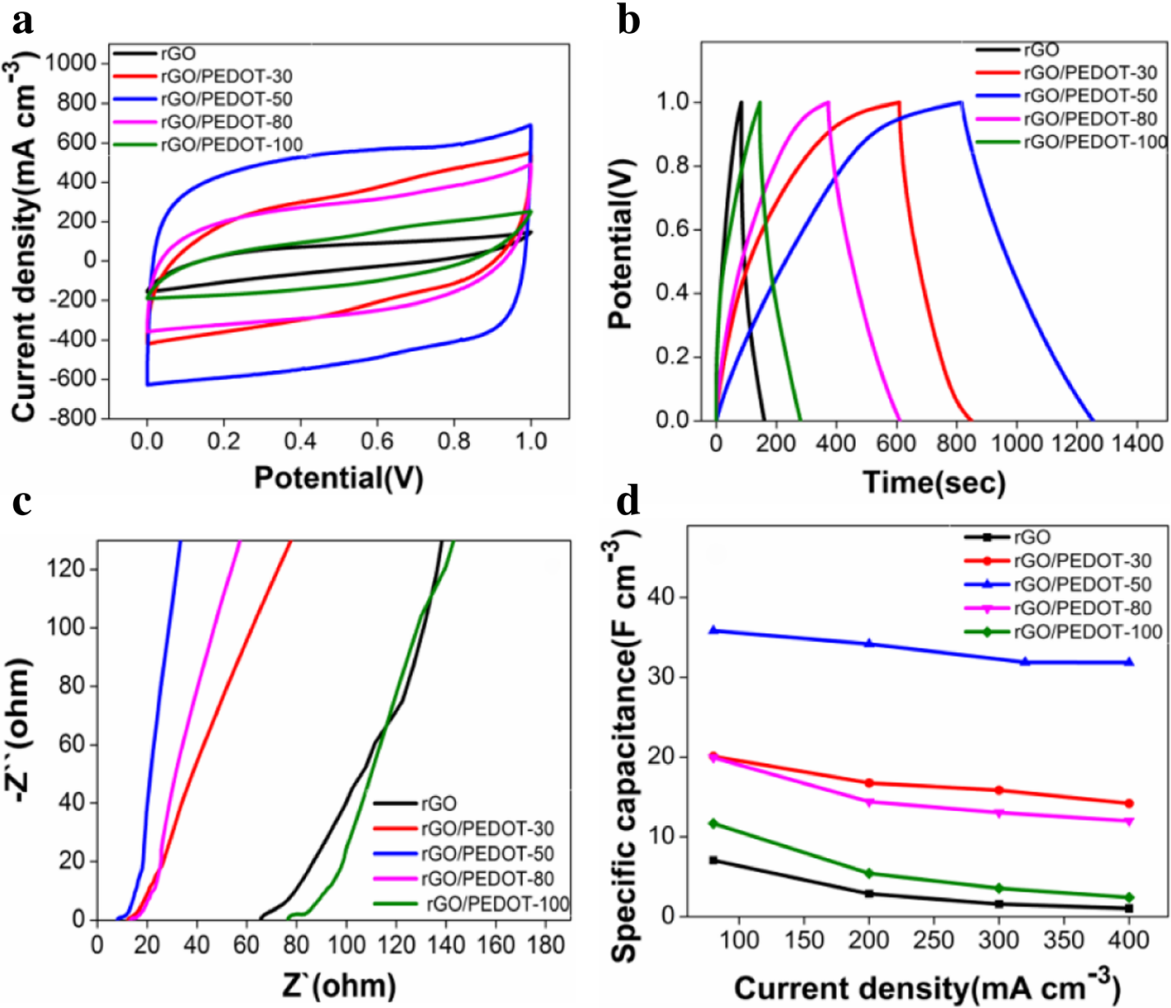
The compared electrochemical properties of various rGO/PEDOT composites with different reaction temperature-based MSCs: **a** CV curves at 20 mV s^−1^ and **b** GCD curves at 80 mA cm^−3^. **c** Nyquist plots from the EIS analysis obtained from 0.01 Hz to 100 kHz. **d** Specific capacitance versus different current densities

The as-prepared porous rGO/PEDOT electrodes can be conveniently assembled into the flexible planar MSCs with PVA/H_3_PO_4_ gel electrolyte, without any conductive additives or binders as well as obtaining simplified and lightweight energy storage devices. In order to evaluate the performance of rGO/PEDOT-based MSCs, their electrochemical properties (Fig. 5) were subsequently investigated via CV, GCD, and EIS measurements using a two-electrode configuration. Figure 5a displays the representative CV plots of rGO/PEDOT-30, rGO/PEDOT-50, rGO/PEDOT-80, rGO/PEDOT-100, and pristine rGO-based MSCs at 20 mV s^−1^. Among them, the CV curve of rGO/PEDOT-50-based MSCs shows a largest quasi-rectangular area, indicating its ideal capacitive behavior. Also, the comparison of GCD curves at 80 mA cm^−3^ was presented in Fig. 5b, which show almost triangular shapes and the potential is nearly linear to charge/discharge time [21]. Impressively, the rGO/PEDOT-50-based MSCs endure the longest discharge time than those for other samples. Additionally, the Nyquist plot of rGO/PEDOT-50-based MSCs (Fig. 5c) shows a nearly vertical profile in the low-frequency region and the smaller inner impedance compared with other samples. Furthermore, the specific capacitances calculated according to the Eqs. (1)–(3) versus discharge current density are shown in Fig. 5d. The corresponding specific capacitance of rGO/PEDOT-50-based MSCs was revealed about 35.12 F cm^−3^ at 80 mA cm^−3^, the specific capacitance displays a gradual drop with increasing the current density, but it still can deliver a relatively high capacity of 31.04 F cm^−3^ at 400 mA cm^−3^ compared to the other four samples, further proving its excellent rate capability.

**Fig. 6:**
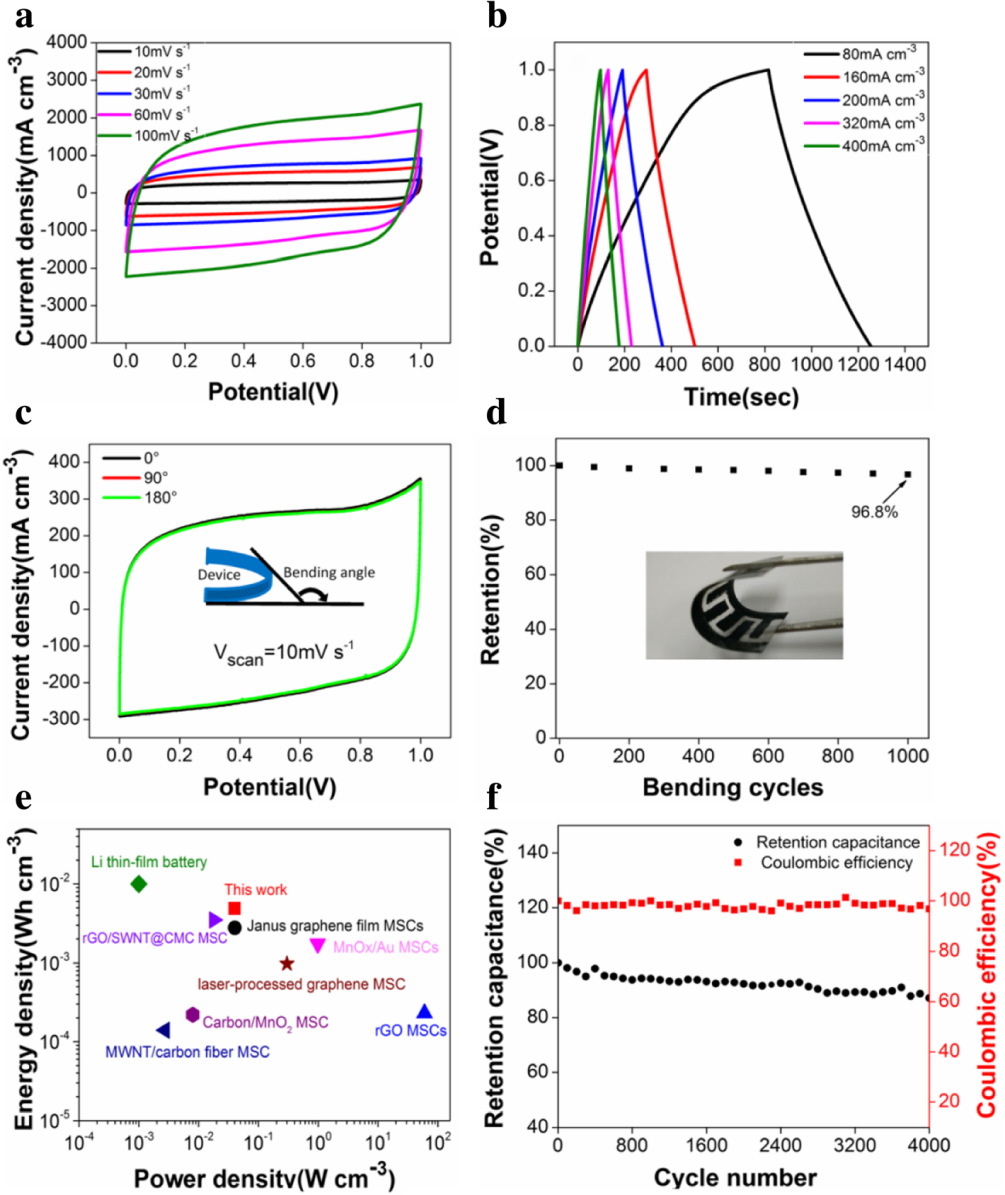
The electrochemical performances of rGO/PEDOT-50-based flexible all-solid-state MSCs: **a** CV curves at various scan rates; **b** GCD curves at different current densities; **c** CV curves obtained under different bending angles at 10 mV s^−1^; **d** Capacitance retention as a function of bending cycles at a current density of 80 mA cm^−3^; **e** Ragone plots of the device and some other reported MSCs, and **f** cyclability tests and coulombic efficiency over 4000 charge/discharge cycles at a current density of 80 mA cm^−3^

To further explore the feasibility of rGO/PEDOT-50-based MSCs, their electrochemical performances were evaluated in Fig. 6. The CV curves retain nearly rectangular shapes with the increasing scan rates from 10 to 100 mV s^−1^ (Fig. 6a), which practically stems from the reversible surface redox reactions of PEDOT and surface electroadsorption of rGO, resulting in the fast charge/discharge rate and the ideal capacitive behavior [38]. Additionally, Fig. 6b shows GCD curves at different current densities under the potential window of 0~1 V, and the nonlinear slopes and triangle shape particularly at lower current densities corroborate the contribution of the pseudo-capacitance from PEDOT, which agrees well with recent reports [39, 40]. Furthermore, the flexibility tests of the planar rGO/PEDOT-50-based MSCs were performed at different angles (Fig. 6c), and the CV curves at 10 mV s^−1^ were almost overlapped under bending with increasing bending angles from 0° up to 180°. Subsequently, the MSCs were bent at 180° for 1000 cycles by a linear motor, and the specific capacitance calculated from the charge/discharge curves retention of 96.8% was achieved after 1000 bending cycles (Fig. 6d). So our MSC devices possess excellent mechanical flexibility, which is mainly ascribed to the flexible PET substrate and the strong adhesion of the 3D highly porous structure with the substrate [41]. These results also confirm the excellent synergistic effect between the laser reduction rGO and VPP polymerized PEDOT. For a micro-device, the energy density and power density are the two critical factors to evaluate its practicality. Therefore, the Ragone plots of as-prepared MSCs and comparison with some other previously reported MSCs are plotted in Fig. 6e. The flexible planar rGO/PEDOT-50-based MSCs deliver a maximum energy density of 4.876 mWh cm^−3^ at a power density of 40 mW cm^−3^, and with the evidence that still remains 4.422 mWh cm^−3^ at 200 mW cm^−3^. These obtained results are comparable or higher than other recent reported MSCs with PVA-based aqueous gel electrolyte such as Janus graphene film MSCs [42], rGO MSCs [28], MnOx/Au MSCs [43], Li thin-film battery [44], MWNT/carbon fiber MSC [45], rGO/SWNT@CMC MSC [46], carbon/MnO_2_ MSC [47], or laser-processed graphene MSC [48]. The cyclability tests and coulombic efficiency of rGO/PEDOT-50-based MSCs over 4000 charge/discharge cycles at a current density of 80 mA cm^−3^ are shown in Fig. 6f. It can be seen that the volumetric specific capacitances keep stable with the retention capacitance of 90.2% after 4000 cycles, and the coulombic efficiencies keep 97~99% during the whole cycles, demonstrating the excellent durability and reversibility of rGO/PEDOT-50-based MSCs.

**Fig. 7:**
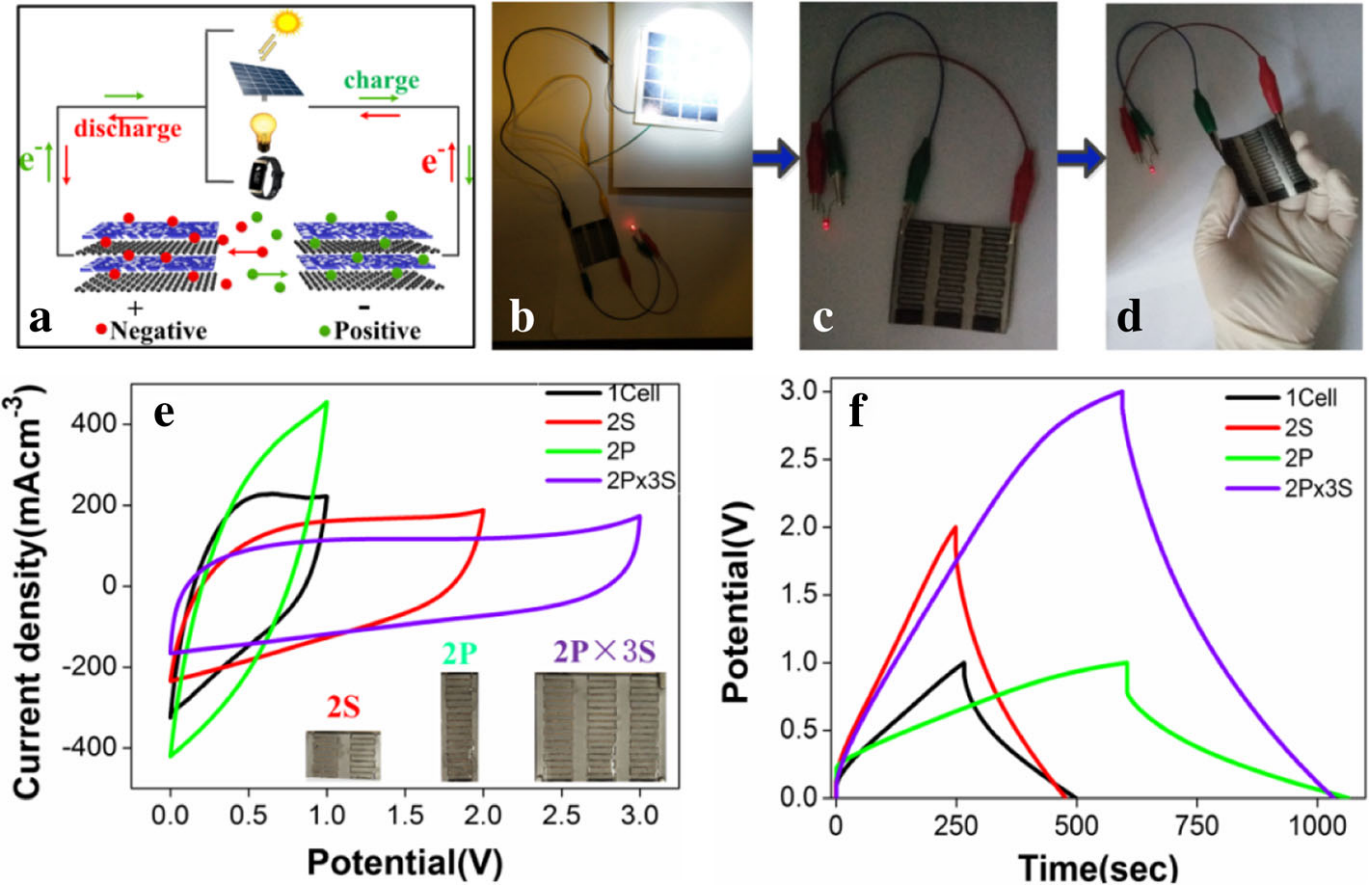
Fabrication of rGO/PEDOT-50-based MSC array as micro-energy storage devices. **a** Schematic of showing the working principle of MSC array integrated with miniaturized electronic devices. **b**~**d** Integration of a flexible MSC array with solar cells for lighting a LED. **e** The CV curves at 20 mV s^−1^ and **f** GCD curves at 40 mA cm^−3^ of MSC array connected in series (2 cells in series, 2S), in parallel (2 cells in parallel, 2P), and in a combination of series and parallel (2 parallel × 3 series, 2P × 3S). The optical images of MSC array insert in **e**

In general, the working voltage, electric current, or capacitances of a single MSC device are too low to meet the demands of miniaturized electronic devices [49]. Therefore, the rGO/PEDOT-50-based MSC array connected in series/parallel was fabricated (Fig. 7) via the cost-effective laser treatment and readily scalable VPP method. Figure 7a shows the moving path of the electrolyte ions along the planar surface of the MSC array integrated with miniaturized electronic devices. Figure 7b–d show a self-powered system integrated a flexible MSC array with solar cells, which is successfully proved by lighting a LED under the deformation state of MSC array. Figure 7e and f show the CV curves at 20 mV s^−1^ and GCD curves at 40 mA cm^−3^ of MSC array, respectively. And the optical images of the assembled MSC array were inserted in Fig. 7e. Especially, the voltage window of MSC array connected in 2P × 3S was expanded up to 3 V, three times higher than that of a single MSC (Fig. 7e), while the charge/discharge time is approximately double of a single device (Fig. 7f), which indicates that the MSC array roughly obeys the basic rules of series/parallel connections [17], and the energy densities of MSC array connected in 2P × 3S were increased by six times comparing to a single MSC. These superior electrochemical performances of rGO/PEDOT-based MSC array owes much to the following possible factors: (1) the interdigitated structures enable the electrolyte ions a higher ion diffusion coefficient as well as shorten planar ionic diffusion path, resulting in further improving their rate capability [41]. (2) The reaction temperature was optimized and the PEDOT direct-growth on rGO at 50 °C by VPP can supply a strong adhesion between their interfacial contact, thus endowing a good electron pathway and enhancing electrochemical durability. (3) The synergistic effect of 3D highly porous structure PEDOT and silk-like rGO (shown in Fig. 2) leads to a large surface area, massive exposed electrochemical reaction active sites, the accessibility to the electrolyte ions, and lowers charge-transfer resistance [50, 51]. Benefiting from the above advantages, the rGO/PEDOT-based MSCs exhibit excellent energy storage characteristics, making them promising micro-energy devices in miniaturized electronic applications.

## Conclusions

In summary, we provide a feasible strategy to conveniently prepare the MSC array with 3D opened network of rGO/PEDOT interdigital electrodes using the laser treating and VPP methods. Interestingly, the required working potential or electric current in most practical applications could be easily tailored by connecting in series/parallel without additional voltage balance management. The obtained rGO/PEDOT-50-based planar interdigital MSCs deliver the high specific capacitance of 35.12 F cm^−3^ (the corresponding energy density of 4.876 mWh cm^−3^) at 80 mA cm^−3^, stable cycling stability (90.2% for 4000 cycles), superior rate capability, excellent coulombic efficiency (keep 97~99% during the whole cycle), and good flexibility under different bending angles. Considering the convenient fabrication, high performances, excellent size compatibility, and flexibility, the rGO/PEDOT-based MSC array is particularly a promising candidate for the next-generation high-performance flexible micro-energy sources integrated with microelectronic devices.

## Data Availability

The datasets used and/or analyzed during the current study are available from the corresponding author on reasonable request.

## Abbreviations

2D: Two-dimensional
3D: Three-dimensional
CV: Cyclic voltammetry
CVD: Chemical vapor deposition
EIS: Electrochemical impedance spectroscopy
Fe (PTS)_3_: iron (III) p-toluenesulfonate
FTIR: Fourier transform infrared spectroscopy
GCD: Galvanostatic charge/discharge
LED: Light-emitting diode
MSCs: Microsupercapacitors
PET: Polyethylene terephthalate
PVA: Polyvinyl alcohol
rGO/PEDOT: Reduced graphene oxide/poly(3,4-ethylenedioxythiophene)
SEM: Scanning electron microscope
VPP: Vapor phase polymerization
XPS: X-ray photoelectron spectroscopy
